# Body weight, egg production, and egg quality traits of gray, brown, and white varieties of Japanese quail (*Coturnix coturnix japonica*) in coastal climatic condition of Odisha

**DOI:** 10.14202/vetworld.2016.832-836

**Published:** 2016-08-10

**Authors:** Jessy Bagh, B. Panigrahi, N. Panda, C. R. Pradhan, B. K. Mallik, B. Majhi, S. S. Rout

**Affiliations:** 1 Department of Livestock Production and Management, College of Veterinary Science and Animal Husbandry, Orissa University of Agriculture and Technology, Bhubaneswar, Odisha, India; 2 Department of Animal Nutrition, College of Veterinary Science and Animal Husbandry, Orissa University of Agriculture and Technology, Bhubaneswar, Odisha, India; 3 Central Poultry Development Organization, Eastern Region, Bhubaneswar, Odisha, India

**Keywords:** body weight, egg production potential, egg quality traits, quail

## Abstract

**Aim:**

The present study was conducted to evaluate the performance of gray, brown, and white varieties of Japanese quail (*Coturnix coturnix japonica*) with respect to body weight, egg production, and egg quality traits in the coastal climatic condition of Odisha.

**Materials and Methods:**

A total of 500-day-old straight run Japanese quail chicks of three varieties, *viz*., gray, brown, and white were randomly selected and reared in deep litter system at Central Poultry Development Organization, Eastern Region, Bhubaneswar. The weekly body weight of the birds was recorded till their egg production stage (up to 6 weeks of age). The average egg production was recorded every biweekly from 6^th^ to 20^th^ week. Exterior and interior quality of eggs from each variety was determined at 6 weeks of age.

**Results:**

The initial average weekly body weight of three varieties did not differ (p>0.05) among the varieties. However, from 1^st^ to 6^th^ week significantly higher body weight was observed in gray than white and brown. Brown varieties had reached 50% egg production 1 week earlier than gray and white. Brown had higher peak hen day (HD) production or hen-housed egg production followed by white and gray. External quality such as: Egg weight, egg length, egg width, volume, shape index, shell weight, shell thickness depicted no significant difference among the varieties except circumference length and circumference width, which were significantly higher (p≤0.05) in gray varieties than brown varieties. Internal egg characteristics such as: Albumen length, albumen width, albumen height, albumen index, yolk length, yolk width, yolk height, yolk index, albumen weight, yolk weight, Haugh unit revealed no significance difference among the varieties.

**Conclusion:**

It may be summarized from the findings that gray excelled in body weight followed by white and brown. Egg production potential in terms of hen house egg production or HD egg production was higher for brown followed by white and gray in the coastal climatic condition of Odisha.

## Introduction

The Japanese quail (*Coturnix coturnix japonica*) is emerging as a promising poultry to the resource-poor rural farmers of India for minimal capital and managemental requirements in rearing. It is the smallest farmed avian species [1] and becoming popular in commercial poultry sector for meat and egg production. Raising quail provides a resource for poor families with meat and eggs [2]. The females are very prolific because they begin laying eggs on average at 6 weeks and can lay between 250 and 300 eggs a year [3]. Furthermore, the quail is an efficient converter of feed with each egg a female deposits an edible package of 8% of her own body weight as compared to 3% in case of chicken [4].

Egg quality is the more important price contributing factor in table and hatching eggs [5]. Therefore, the economic success of a laying flock solely depends on the total number of quality eggs produced [6]. Besides the performance of three varieties, the present study would be helpful to express a preference for the appropriate one in this situation.

Although a lot of works have been carried out on the performance of egg production potential and egg quality traits of quail, it is desirable to know the performance of the above-mentioned varieties under hot and humid conditions of Odisha, the information on which is limited.

## Materials and Methods

### Ethical approval

Experiments were carried out following the guidelines of the Institutional Animal Ethics Committee.

### Location and period of experiment

The experiment was carried out at the Central Poultry Development Organization (CPDO), Government of India, Eastern Region, Bhubaneswar. The experiment was conducted during the period starting from January to May 2014.

### Experimental programme

A total of 500-day-old straight run thrifty and healthy Japanese quail chicks of three varieties, *viz*., gray, brown, and white were randomly selected and reared in deep litter system at CPDO, Eastern Region, Bhubaneswar. The experimental birds were placed in the naturally ventilated house and were raised in the deep litter system. The feed and water were given *ad libitum* and fed with conventional starter, grower and layer rations, respectively. Natural daylight was provided at the beginning of the experiment and then light hour was increased by ½ h/day. Vaccination and preventive medication were followed as per standard procedure during the experimental period. There was provision of one nest box with three compartments for every 20 laying quails in the layer house for ensuring clean egg production from the farm was determined according to the method of Debata *et al*. [7]. Average age at sexual maturity of the three varieties was taken by recording the age at first egg production of all the birds. The average egg production mainly from 6^th^ to 20^th^ week, i.e., 15 weeks interval was recorded every biweekly. Then, cumulative egg production up to 20 weeks was also calculated. External and internal egg quality of 10 freshly laid eggs from each of the three varieties of quail was taken for the study at 6 weeks of age.

### Measurement of external egg quality

Egg weight was individually recorded on a digital balance to the nearest of 0.01 g accuracy. Egg length (along the longitudinal axis) and egg width (along the equatorial axis) were measured with the help of digital calipers to the nearest to 0.01 mm. Shape index of the individual egg was calculated as the ratio of maximum egg width to length (%) as per Schuttz [8]. Volume of the egg was measured by liquid displacement technique using the 1 L graduated cylinder setup. Circumference length and circumference width were measured by measuring tape. After the eggs were broken, egg shells were washed under slightly flowing water to remove remaining albumen and were left to dry at room temperature for 24 h; then, they were weighed in the digital balance. Shell thickness along with membrane was measured at the broad end, narrow end, and equatorial parts of each egg to the nearest of 0.01 mm with the help of screw gauze and shell thickness was obtained from the average value.

### Measurement of internal egg quality

With the help of a Vernier caliper, the length and width of the thick albumen and yolk were measured in millimeter. The height of the thick albumen and yolk was measured at maximum height with the help of spherometer to 0.01 mm accuracy on a leveled glass table. The albumen index and the yolk index were worked out according to the formula of Heiman and Carver [9] and Sharp and Powell [10], respectively. Haugh unit was calculated according to the formula of Haugh [11]. The yolk separated from the albumen was weighed together with the membrane to get the yolk weight [12]. Albumen weight was calculated by subtracting the yolk and shell weight from egg weight.

### Statistical analysis

The data obtained from the study were statistically analyzed according to Snedecor and Cochran [13]. The data were analyzed for analysis of variance to test the difference between means wherever necessary.

## Results

### Body weight

The average weekly body weight of the three varieties from day old to 6 weeks is presented in the Table-1. The initial body weight of three varieties ranged from 7.13 to 7.50 g, which did not differ significantly (p>0.05) among the varieties. In the 6^th^ week, the body weight of gray, brown, and white was 173.79, 168.23, and 172.62 g, respectively. Higher body weight was observed in gray than the white and brown in a 6^th^ week. It was also seen that brown varieties have less body weight than white. As the quails under the study attain sexual maturity around 5-6 weeks, so the body weights were recorded up to 6^th^ week.

**Table-1:** Weekly body weight (g) of three varieties of Japanese quail.

Age (weeks)	Gray	Brown	White	SEM
0	7.50±0.15	7.38±0.12	7.13±0.13	NS
1	18.37^c^±0.16	20.26^b^±0.18	21.63^a^±0.19	0.02
2	48.86^a^±0.38	43.97^c^±0.38	47.95^b^±0.37	0.04
3	92.26^a^±0.67	84.37^c^±0.73	91.34^b^±0.80	0.08
4	120.43^b^±0.78	112.98^c^±0.92	134.46^a^±0.50	0.07
5	152.80^a^±0.90	146.16^c^±0.97	149.05^b^±0.87	0.20
6	173.79^a^±1.01	168.23^c^±0.95	172.62^b^±1.25	0.30

Means showing different superscripts in a row differ significantly (p<0.05). SEM: Standard error of mean, NS: Not significant

### Egg production

The hen-housed (HH) egg production and hen day (HD) egg production of three varieties from 6 to 20 weeks are presented in Table-2. Brown varieties had reached 50% egg production 1 week earlier than gray and white. Brown had higher (99.7%) peak HD production followed by white (89.2%) and gray (87.3%). The hen-housed egg production (%) over time followed the same pattern as observed for hen day egg production (%). Table 2

**Table-2:** HH and HD egg production (%).

Age in weeks	HH egg production (%)			HD egg production (%)		
	
	Gray	Brown	White	Gray	Brown	White
6	0.00	0.00	0.33	0	0	0.33
7	16.08	20.80	19.23	16.14	20.80	19.2
8	61.78	80.70	57.58	62.49	83.65	58.9
9	80.70	96.12	67.91	81.63	99.63	69.5
10	91.30	96.12	86.92	92.35	99.63	88.9
11	86.38	96.24	87.25	87.37	99.76	89.2
12	86.38	96.24	87.25	87.37	99.76	89.2
13	81.08	95.86	84.84	82.01	99.37	86.8
14	82.78	95.24	80.22	83.74	98.72	82.0
15	82.88	88.72	83.96	85.05	94.03	86.3
16	64.62	81.95	72.42	66.31	87.67	75.0
17	63.58	77.69	72.64	65.69	83.68	75.2
18	67.74	84.71	77.36	69.99	91.23	80.1
19	71.24	75.06	76.92	73.61	80.84	79.6
20	65.00	80.45	73.41	67.16	86.65	76.0

HH: Hen house, HD: Hen day

### External egg characteristics

As far as the external egg qualities of the egg are concerned, the circumference length and circumference width were significantly higher (p≤0.05) in gray varieties than brown varieties, and no difference was seen between gray and white varieties (Table-3). The egg length, egg width, volume, and shape index of these varieties depicted no significant difference among themselves.

**Table-3:** External egg quality traits of three varieties of Japanese quail.

Parameters	Gray	Brown	White	SEM
Egg weight (g)	11.6±0.03	10.58±0.30	10.58±0.26	NS
Egg length (mm)	32.51±0.33	31.43±0.23	31.43±0.40	NS
Egg width (mm)	25.83±0.88	24.74±0.24	24.33±0.27	NS
Circumference length (mm)	9.53^b^±0.40	8.79^c^±0.11	9.69^a^±0.06	0.04
Circumference width (mm)	8.37^a^±0.08	7.76^b^±0.14	8.42^a^±0.2	0.05
Volume (ml)	10.40±0.37	9.70±0.26	10.10±0.31	NS
Shape index (%)	72.88±3.14	72.13±0.88	69.93±1.51	NS
Shell weight (g)	1.56±0.01	1.42±0.02	1.5±0.06	NS
Shell thickness (mm)	0.26±1.61	0.27±2.76	0.25±0.01	NS

Values bearing different superscripts in a row differ significantly (p<0.05). SEM: Standard error of mean, NS: Not significant

### Internal egg characteristics

Albumen length, albumen width, albumen height, albumen index, yolk length, yolk width, yolk height, yolk index, albumen weight, Haugh unit, and yolk weight are presented in Table-4. The values were not significantly different (p≥0.05) between gray, brown, and white varieties.

**Table-4:** Internal egg quality traits of three varieties of Japanese quail.

Parameters	Gray	Brown	White	SEM
Albumen width (mm)	32.73±0.65	35.32±1.25	33.49±0.76	NS
Albumen length (mm)	43.34±1.46	44.80±1.51	45.92±1.77	NS
Albumen height (mm)	4.76±0.01	4.73±0.01	4.66±0.23	NS
Albumen weight (g)	5.20±0.17	4.78±0.19	5.40±0.12	NS
Albumen index	14.10±0.16	13.20±0.52	13.15±0.01	NS
Haugh unit	91.01±1.57	91.28±1.71	90.90±1.20	NS
Yolk length (mm)	24.57±0.32	24.81±0.51	25.23±0.64	NS
Yolk width (mm)	23.11±0.25	23.76±0.32	23.90±0.86	NS
Yolk height (mm)	11.15±0.01	10.86±0.01	10.66±0.31	NS
Yolk weight (g)	3.80±0.06	3.42±0.08	3.50±0.13	NS
Yolk index	48.05±0.22	45.25±0.31	45.36±0.03	NS

SEM: Standard error of mean, NS: Not significant

## Discussion

### Body weight

In the present study, the white had higher body weight in 1^st^ week than brown and gray, but from 2^nd^ to 6^th^ week, gray had higher body weight which could be attributable to the genotype for higher rate of gain of the variety. These findings are in line with those of Vali *et al*. [14], who reported significant body weight variation in two quail strains at 35, 42, and 49 days of age. Strain effect on body weight at different ages is reported by different authors Aljumaily [15] and Jatoi *et al*. [16]. Aljumaily [15] reported that brown had higher body weight than white strain at 2, 3, 4, and 5 weeks of age (p<0.05), which is not in agreement of present study finding.

### Egg production

In present study, brown birds had more HH egg production than gray and white, where brown birds are lighter than gray and white. Similarly, Jatoi *et al*. [16] in another study with 4 different close-bred (three local and one imported) Japanese quails reported that the maximum egg production was recorded in the small weight category and minimum in the heavy size birds. Similar findings have also been observed by North and Bell [17] and Ipek *et al*. [18] in poultry birds. Findings are in quite agreement with those of Nestor and Bacon [19] indicating that egg production decreased in heavy size and increased in low body weight strains of Japanese quail. High egg production in small quails in comparison to heavy quails could be due to more number of mature ovarian follicles in small quails. Jatoi *et al*. [16] also reported the similar findings.

### External egg characteristics

Significantly higher (p≤0.05) circumference length and circumference width of gray and white varieties than brown varieties might be ascribed to the higher body weight of gray and white birds and lower egg production efficiency. This is in agreement with the findings of Hrncar *et al*. [20] that eggs of egg type Japanese quail weighed 11.48 g, whereas the eggs of meat type Japanese quail weighed 13.06 g and also found that egg width (mm) were 25.71 and 21.94, egg length (mm) were 33.52 and 34.46, egg shape index (%) were 76.70 and 78.18, shell weight (g) were 1.02 and 1.16, and shell thickness (mm) were 0.25 and 0.23, for egg type and meat type quails, respectively. Zita *et al*. [21] calculated the egg weight and egg shape index in Pharaoh strain meat type quails as 12.52 g and 77.85%, respectively similar to results obtained in the present experiment.

### Internal egg characteristics

An experiment conducted by Hrncar *et al*. [20] found that albumen weight was 6.75 g and 7.52 g, albumen length was 50.14 and 49.82, and albumen width was 38.27 and 38.27. Haugh unit was 87.28 and 87.56 in egg type and meat type quails, respectively. Due to the small size of eggs in the present experiment, the corresponding values obtained for albumen weight, albumen length, albumen width, and albumen height were less compared with the Hrncar *et al*. [20]. The Haugh unit values in this study (90.90, 91.01, and 91.28) were in agreement with the data reported in literature for Haugh unit, such as 85.53-95.21 in quail by Altan *et al*. [22]. As for the yolk quality traits, the present study has shown a non-significant difference among the varieties. The values for yolk quality traits in this study were in agreement with the findings of Zita *et al*. [21]. The higher the Haugh unit and yolk index, the more desirable is the interior quality of the egg [23].

## Conclusion

From this, it may be concluded that gray excelled in body weight followed by white and brown. Egg production potential, in terms of HH egg production or HD egg production, was higher for brown followed by white and gray.

## Authors’ Contributions

This present study is the major component of the work toward the M.V.Sc. thesis of the first author JB. JB carried out the work under the guidance of BP, NP, BKM, and CRP. BM and SSR: Helped during the study. NP guided in the statistical analysis of data. BP and CRP participated in draft and revision of the manuscript prepared by JB. All authors read and approved the final manuscript.
